# SARS-CoV-2/influenza A (H3N2) virus coinfection: epidemiological surveillance in Northeast Brazil

**DOI:** 10.1590/0037-8682-0132-2022

**Published:** 2022-08-05

**Authors:** Cliomar Alves dos Santos, Gabriela Vasconcelos Brito Bezerra, Aline Rafaelle Rocha Almeida de Azevedo Marinho, Ludmila Oliveira Carvalho Sena, Vitoria de Jesus Menezes, Daniela Cabral Pizzi Teixeira, Mércia Feitosa de Souza, Marco Aurélio de Oliveira Góes, Paulo Ricardo Martins-Filho

**Affiliations:** 1 Governo do Estado de Sergipe, Fundação de Saúde Parreiras Horta, Laboratório Central de Saúde Pública de Sergipe (LACEN/SE), Aracaju, Sergipe, Brasil.; 2 Governo do Estado de Sergipe, Secretaria de Estado da Saúde, Aracaju, Sergipe, Brasil.; 3 Universidade Federal de Sergipe, Departamento de Medicina, Lagarto, Sergipe, Brasil.; 4 Universidade Federal de Sergipe, Laboratório de Patologia Investigativa, Programa de Pós-graduação em Ciências da Saúde, Aracaju, Sergipe, Brasil.

Dear Editor,

In Brazil, more than 28 million coronavirus disease 2019 (COVID-19) cases and 649,000 associated deaths have been reported as of February 2022. Despite substantial reductions in hospitalizations and COVID-19 deaths and increased vaccine administration, the spread of the omicron variant led to a sharp increase in COVID-19 cases from January to February 2022. In addition, Brazil has faced an outbreak of nonseasonal flu cases caused by a new strain of influenza subtype A (H3N2) named Darwin. In most Brazilian states, the usual flu season is from May to July, and starts in the northeast region and spreads to the South^1^. Identifying the so-called “twindemic” of COVID-19 and the flu is challenging because both infections cause similar respiratory manifestations, and Brazil's testing capacity is limited. In addition, evidence on the prevalence and outcomes associated with severe acute respiratory syndrome coronavirus 2 (SARS-CoV-2) and influenza virus coinfection in Brazil is lacking. 

This descriptive report presents the results of epidemiological surveillance of patients with SARS-CoV-2 and influenza A (H3N2) virus coinfection in the State of Sergipe in northeastern Brazil from December 15, 2021 to January 14, 2022. The state of Sergipe is located in a region with the worst socioeconomic indicators in the country, with an estimated population of 2.3 million. This state has a Human Development Index of 0.665. 

The first case of COVID-19 in Sergipe was confirmed on March 14, 2020 in a woman who traveled to Spain and was infected with SARS-CoV-2 lineage B.1. As of January 14, 2022, the State of Sergipe registered 279,587 confirmed COVID-19 cases and 6064 COVID-19-associated deaths. In addition, complete vaccination coverage against the disease with two doses of CoronaVac (Sinovac Biotech, Beijing, China), Covishield (AstraZeneca, Cambridge, UK), or ComiRNAty (Pfizer- BioNTech, New York, USA) vaccines or a single-dose of the Johnson & Johnson (Janssen) vaccine was estimated at 68%. The first out-of-season case of influenza A virus subtype H3N2 infection was confirmed on December 15, 2021. Approximately 75% of the at-risk and priority groups (older adults, pregnant and postpartum women, healthcare professionals, and children aged 6 months to 6 years) were vaccinated against influenza. Trivalent vaccines including the A/Victoria/2570/2019 (H1N1) pdm09-like virus, A/Hong Kong/2671/2019 (H3N2)-like virus, and B/Washington/02/2019 (B/Victoria lineage)-like virus were administered for flu vaccination in 2021.

All patients included in this analysis had laboratory-confirmed SARS-CoV-2 and/or influenza A virus subtype H3N2. Respiratory samples were analyzed by the Central Laboratory of Public Health, which has been used as the laboratory of reference for the diagnosis of public health diseases in each Brazilian state^2^. Patient demographic data, including age, sex, COVID-19 vaccine status, and mortality were recorded. Case-fatality rates were calculated based on the number of deaths from SARS-CoV-2, influenza A (H3N2), and SARS-CoV-2/influenza A (H3N2) coinfection divided by the total number of confirmed cases for each condition.

From December 15, 2021 to January 14, 2022, a total of 1458 and 1237 COVID-19 and influenza cases were diagnosed, respectively. The case-fatality rates for COVID-19 and influenza were 0.8% (11 deaths) and 3.0% (37 deaths), respectively. Fifty-two cases of SARS-CoV-2 and influenza A (H3N2) virus coinfection were detected during this period; the median patient age was 64 years (1-99 years). Most patients were over 60 years old (57.7%), of which 51.9% were men and 48.1% were women. Thirty-nine (75.0%) patients with coinfections were fully vaccinated against COVID-19: CoronaVac was administered to 22 (56.4%) patients, Covishield to 14 (35.9%) patients, and ComiRNAty to 3 (7.7%) patients. However, 13 patients (25.0%) were unvaccinated or partially vaccinated. Three deaths were recorded, and the case-fatality rate was 5.8%. All deaths occurred among women over 60 years of age who were vaccinated against influenza. However, only two patients were fully vaccinated against COVID-19 (Table 1).

**TABLE 1:** Clinical characteristics of patients with SARS-CoV-2 and influenza A (H3N2) virus coinfection (n = 52)**.**

Parameters	n (%)
Age ≥ 60 years	30 (57.7)
Male sex	27 (51.9)
COVID-19 vaccination status	
Fully vaccinated	39 (75.0)
Partially vaccinated / unvaccinated	13 (25.0)
Deaths	3 (5.8)

**COVID-19:** corona virus disease 2019; **SARS-CoV-2:** severe acute respiratory syndrome coronavirus 2.

SARS-CoV-2 and influenza A (H3N2) coinfection was a major challenge for the healthcare system in late 2021 and early 2022 in Brazil. With the resumption of international tourism and opening of international borders worldwide, Brazil confirmed imported cases of the omicron variant and influenza A (H3N2) virus in December 2021. Moreover, a reduction in public health measures to mitigate the spread of COVID-19, including social distancing, the use of face masks, and school and business closures, have contributed to the advancement of the omicron variant and transmission of influenza in Brazil. However, the prevalence of SARS-CoV-2 and influenza coinfection varies across Brazilian states^3^ owing to differences in vaccine coverage, adherence to nonpharmaceutical interventions, testing capacity, and socioeconomic inequities. This is the first study to investigate mortality data associated with SARS-CoV-2 and out-of-season influenza coinfection in Brazil, although the actual prevalence of individuals with coinfection may be underestimated. Patients with respiratory symptoms must be tested, and those with SARS-CoV-2 and influenza A (H3N2) virus coinfection must be carefully monitored during the influenza season in different Brazilian regions. 

Despite the relatively low prevalence (approximately 2.0%) in the State of Sergipe, our findings add to the growing body of evidence on the severity of SARS-CoV-2 and influenza A (H3N2) virus coinfection. Recent observational studies reported that coinfection with SARS-CoV-2 and influenza A (H1N1) predominantly affected older people and were associated with poorer clinical outcomes^4^ ^,^ ^5^. However, mortality data from meta-analyses showed conflicting results because of between-study heterogeneity^6^ ^,^ ^7^. Our results showed a high case-fatality rate among patients coinfected with SARS-CoV-2 and influenza A (H3N2) virus, even among vaccinated patients. However, the influenza vaccination campaign in Brazil excluded the A/Darwin/9/2021-like strain of the H3N2 component in the vaccines administered in 2021. These findings suggest that patients who are coinfected and nonimmunized develop more severe inflammatory response syndrome and potentially fatal clinical outcomes. On February 25, 2022, the Butantan Institute delivered a batch of 2 million doses of the 2022 flu vaccine to the National Immunization Program, comprised of H1N1, strain B, and H3N2 Darwin. 

This study showed a higher case-fatality rate for SARS-CoV-2 and influenza A (H3N2) coinfection than that observed for isolated infections. Future studies are needed to evaluate the “twindemic” burden in Brazil as well as the related outcomes in other regions. 
